# Supplementary material for: Causal Relationship between Aging and Anorexia Nervosa: A White-Matter-Microstructure-Mediated Mendelian Randomization Analysis
Source: Biomedicines. 2024 Aug 16;12(8):1874. doi: 10.3390/biomedicines12081874 (PMC11351342; doi:10.3390/biomedicines12081874)
Supplement: Supplementary file 1 [file biomedicines-12-01874-s001.zip › Supplementary Figure/Figure S8/Leaveoneout plot of MR analysis for TL to ubm-b-2069.pdf]

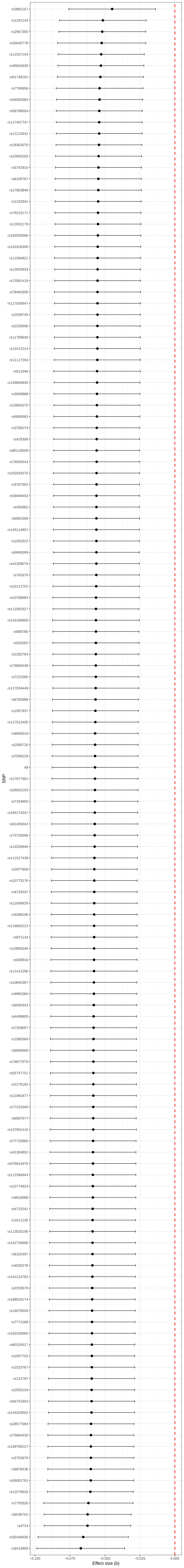
rs3891167
rs1291143
rs2967355
rs35640778
rs11557154
rs45604339
rs61748181
rs7790856
rs34550383
rs56799554
rs117407747
rs11212631
rs28363070
rs10905255
rs5742915
rs8105767
rs17803849
rs1332941
rs76219171
rs12932179
rs182059586
rs142426306
rs11584821
rs12925933
rs73581419
rs78491606
rs117630647
rs2538745
rs2230590
rs11769630
rs12412214
rs11117354
rs611646
rs139669835
rs3093888
rs10840270
rs6669563
rs3785074
rs429358
rs80116508
rs76065543
rs202034370
rs3767952
rs59409453
rs450962
rs6881568
rs145114957
rs1003322
rs9940099
rs41269079
rs762679
rs10112752
rs10768683
rs111950327
rs143190905
rs869785
rs932002
rs2282764
rs76666449
rs7221585
rs117034449
rs6790988
rs1957937
rs117512405
rs9600019
rs2056726
rs7099229
All
rs17677991
rs28502153
rs7164950
rs185174247
rs61405042
rs73730598
rs13230646
rs111527438
rs2977608
rs10773176
rs4743037
rs11699829
rs9398196
rs116863223
rs871134
rs10805346
rs939916
rs11412296
rs10845387
rs9955360
rs6590343
rs4498805
rs7209057
rs1985369
rs6659669
rs79977579
rs55747751
rs2276182
rs11991877
rs77231040
rs6587577
rs137901416
rs77732866
rs41304832
rs376641875
rs112394943
rs10774624
rs4616688
rs4731541
rs1611236
rs113525195
rs142730696
rs8102497
rs4530278
rs141214782
rs2293579
rs188918174
rs16978028
rs7772289
rs150150565
rs80324517
rs1907702
rs1023767
rs131797
rs2555104
rs66731853
rs144204502
rs28577594
rs75664430
rs139795227
rs2763979
rs9878436
rs56061761
rs11579626
rs7705526
rs6536702
rs4724
rs35446936
rs9419958
SNP
−0.100
−0.075
−0.050
−0.025
0.000
Effect size (b)
